# Developing novel anthelmintics from plant cysteine proteinases

**DOI:** 10.1186/1756-3305-1-29

**Published:** 2008-09-01

**Authors:** Jerzy M Behnke, David J Buttle, Gillian Stepek, Ann Lowe, Ian R Duce

**Affiliations:** 1 School of Biology, University of Nottingham, University Park, Nottingham, NG7 2RD, UK; 2 Academic Unit of Molecular Medicine, University of Sheffield Medical School, Beech Hill Road, Sheffield S10 2RX, UK; 3 Institute of Comparative Medicine, Faculty of Veterinary Medicine, University of Glasgow, G61 1QH, UK

## Abstract

Intestinal helminth infections of livestock and humans are predominantly controlled by treatment with three classes of synthetic drugs, but some livestock nematodes have now developed resistance to all three classes and there are signs that human hookworms are becoming less responsive to the two classes (benzimidazoles and the nicotinic acetylcholine agonists) that are licensed for treatment of humans. New anthelmintics are urgently needed, and whilst development of new synthetic drugs is ongoing, it is slow and there are no signs yet that novel compounds operating through different modes of action, will be available on the market in the current decade. The development of naturally-occurring compounds as medicines for human use and for treatment of animals is fraught with problems. In this paper we review the current status of cysteine proteinases from fruits and protective plant latices as novel anthelmintics, we consider some of the problems inherent in taking laboratory findings and those derived from folk-medicine to the market and we suggest that there is a wealth of new compounds still to be discovered that could be harvested to benefit humans and livestock.

## Introduction

Intestinal nematodes are extremely important pathogens of domestic livestock, especially of sheep, goats and cattle. Collectively, they are responsible for severe losses to livestock agriculture throughout the world. It has been calculated that, in the U.K., intestinal worms constitute the most important disease-related cost of farming sheep, being responsible for an estimated annual loss to the industry of £83 million [1]. In developing countries of the world, intestinal worm infections are perceived to be the single most important threat to economic success, as was made dramatically clear in a recent review of the attitudes and concerns of small holder farmers in Africa [2].

Intestinal nematodes are also important pathogens of humans, with a range of pathologies and consequences for human health [3]. Four species dominate: *Ascaris lumbricoides*, *Trichuris trichiura *and the two hookworms *Ancylostoma duodenale *and *Necator americanus*. Global estimates of disability adjusted life years lost to infection are almost 5 million with some 3 billion people around the world believed to carry some of the species involved [3,4].

## Synthetic anthelmintics and resistance

The treatment of intestinal nematode infections in the 21^st ^century is largely through the use of modern synthetic anthelmintics. Three classes of these anthelmintics dominate the market, each mediating its effect through a different mode of action on the target nematodes. The group 1 anthelmintics include the benzimidazoles and these were introduced in the early 1960s for use in livestock but resistance (in this case to thiabendazole) was detected after only 4 years of usage in the U.S.A. The group 2 anthelmintics, the nicotinic acetylcholine agonists such as pyrantel, levamisole, morantel etc., were introduced in the early 1970s and resistance was detected for the first time in 1977 in Australia. The macrocyclic lactones (e.g. ivermectin), which form the group 3 anthelmintics, were first licensed for use in the early 1980s and resistance became apparent again within seven years and was first reported in S. Africa [5].

Since then, resistance has spread around the globe, particularly in species affecting sheep, but it is also a significant problem in the husbandry of horses, especially with respect to the cyathostomins [5-7], and is an increasing problem in cattle and pigs [8,9]. Of particular concern is the discovery of triple resistant nematodes which cannot be easily controlled by any of the three classes of drugs. This was first detected in S. Africa in sheep, and then in Scotland among Angora goat flocks [10], but is now known to be more widely distributed [11].

There are also some indications that human hookworms, notably *Necator americanus *and *Ancylostoma duodenale *are becoming less sensitive to the benzimidazoles and to pyrantel, respectively [12-14]. Studies in communities in Tanzania have shown that the efficacy of mebendazole has declined significantly in areas where mebendazole was given routinely to school aged children in a national program to reduce GI nematode infections in the population and to give the children a good start in life [15,16]. Currently, there are a number of new programs being implemented in developing countries based on mass treatment of populations with ivermectin or praziquantel, each combined either with mebendazole or albendazole, and these have already made a significant impact on the prevalence of intestinal parasitic infections in these regions [17-19]. Nevertheless, veterinary parasitologists have repeatedly warned those working in the medical profession that where mass delivery of anthelmintics is being implemented, the lessons from the veterinary experience should be taken on board by the medical profession and, if we are to preserve the efficacy of the available drugs into the future, appropriate actions should be taken before it is too late [20,21].

Although there are signs that novel synthetic drugs are being developed (e.g. nitazoxanide, cyclic depsipeptides, octadepsipeptides such as emodepside, tribendimidine, diketopiperazines such as paraherquamides, amino-acetonitrile derivatives {AADs}), no new drugs that operate through a different mode of action to the Class 1, 2 and 3 anthelmintics have become available on the market for the treatment of either livestock or humans [22-26]. However, there is an exception for companion animals because emodepsides (a product of fungi associated with the leaves of *Camellia japonica*) are being marketed already for treatment of worms in cats (Bayer Health Care under the trade name Profender). Nevertheless, it is now 28 years since the avermectins appeared, and we are still largely constrained by having to rely on just three classes of anthelmintics, so despite the promise of new agents in the pipeline, the range of effective agents is extremely limited and there is a pressing need for new/additional solutions to the problem of controlling intestinal worm infections. The obvious route to follow is to develop novel synthetic drugs. In this context, it may be that as yet undeclared alternative drugs have been developed already by pharmaceutical companies but assessed as uncompetitive in the current market in the face of the overwhelming success of the macrocylic lactones. It is possible that some such drugs are being kept in the wings until they have a likelihood of success, and can take centre stage, when the current agents become totally redundant. Whatever the explanation for the lack of overt progress on this front, whether through failure to develop novel compounds or failure to develop commercially competitive agents, the lack of new drugs over the last 28 years indicates that it is not easy in this day and age to develop novel drugs with a different mode of action from those already on the market.

## Medicinal plants and the problems of developing their active principles as novel medicines

An alternative is to exploit naturally-occurring compounds that exist in plants and trees and in their seeds and fruits. Medicinal plants and fruits have been used by indigenous peoples for centuries as sources of extracts used in the treatment of a variety of problems, including infectious diseases and those caused by parasites, in livestock and humans [27-29]. These are often referred to as ethno-veterinary or ethno-medical remedies, and, in general, they are shunned by traditional, conventional western medicine.

There have been considerable efforts to identify the active ingredients and indeed some are well known [30-32], but, to a large extent, few have lived up to their expectations when tested rigorously [33], and even fewer have been found to have curative properties that can compete effectively with synthetic drugs [27]. Nevertheless, some exceptional natural products, have become widely accepted. Quinine for the treatment of malaria is an obvious example, as is artemisinin or quinghaosu from *Artemisia annula *[30,34]. Penicillin is a fungal product and indeed ivermectin itself is a bacterial product, being derived from the soil bacterium *Streptomyces avermitilis *[35]. Among plant derived molecules that have anti-parasitic activity and have been used as veterinary parasiticides at times there are nicotine, pyrethrum and rotenone. The former two provided templates for the development of synthetic mimics which include the pyrantel group for nematodes and synthetic insecticides, respectively. However, to our knowledge, there are no naturally-derived plant products sold commercially in the markets of the developed nations of the world for the treatment of worm infections [31].

If natural plant products are to be exploited in the future as medicines for human use or for the treatment of livestock, then isolation and characterisation of their active principles becomes an essential prerequisite to further progress. Problems which follow include development of methods for their stabilisation, preservation, formulation, delivery etc., and all this requires enormous development costs and many years of patient work. Furthermore, the necessary clinical trials of efficacy (randomised, double-blind, placebo-controlled) and essential safety trials add, to the delay before drugs become available for treatment, and enormously to the costs sustained by developers. Inherent in the system is the accompanying bureaucracy required by authorities to licence novel drugs for commercial production and this adds yet another tier of complexity, expense and delay.

Perhaps the most important impediment to further progress is the obstacle presented by "prior art". Although contemporary laboratory techniques may help to identify the active compounds and solve some of the initial problems referred to above, the ultimate developmental costs are dependent on investment by industry. But pharmaceutical firms are not charities. Their priority is to ensure good returns on their outlay, for reinvestment in research and for their shareholders; profits usually facilitated by patents protecting the key steps involved in extraction or manufacture of the active principles. For many medicinal plants, there is already too much published information in the public domain to enable compounds to be patented securely, although if structure has not been disclosed, patenting is possible. Moreover, it is very clear to us that there is a conflict between disclosure as part of academic research (publish or die!) and secrecy to ensure patentability in the longer term. These are real obstacles to investment by industry, and an anomaly of the system that is hindering progress in a world where novel drugs are urgently required. It is also a sad reflection of our priorities since it is very clear that there are active compounds that could be developed into new generations of modern medicines. If we are to make progress, trusts and public funds will need to support the initial sequence of developmental steps, possibly right through to the marketable product and at least some such schemes have already been initiated (e.g. Wellcome Trust Translational Awards Scheme). By way of encouragement, it is worth pointing out that in developing formulations and delivery systems, it is conceivable that novel steps will be discovered and these will allow patents to be filed eventually and industry to be guaranteed protection.

There are of course other problems that deter pharmaceutical companies from exploiting plant derived medicines. These include variability in the concentration of active principles in relation to how the plants were grown, climate, soil quality, site, and season of the year. Additionally governments may charge fees to access sources of relevant plants, and royalties for their exploitation, and these charges may eat into profits, discouraging investment and development.

An alternative avenue might be to develop natural products as food additives. Concerns about residues in food and animal welfare under intensive stocking densities have stimulated a growing market in the developed world for organic farming, and organically farmed livestock are as susceptible to parasites, perhaps even more so than those intensively reared and regularly drenched with wormers. Whole plants, leaves, stems, fruits and seeds containing activity against worms could be incorporated into feed without loss of organic status. This would be more acceptable where the whole plant is itself a suitable food for the target animals, as in the case of fruits, but perhaps less so where the only benefit of eating would be from the medicinal principles, since plants, like all biological material, are extremely complex and may contain thousands of molecules, only some of which would bring benefits while others may be toxic. A good example of the use of fodder that does not support transmission of intestinal worms is in chicory on organic sheep farms [36], or plants with high tannin content [37]. More refined methods of administration may include extracts from plant tissues, given on their own or in combination with conventional fodder.

Indeed, this whole approach may spawn a novel industry, searching for novel plant varieties with up-regulated content of the active principles. In this age of genetic manipulation, transgenic approaches may also pay dividends [32], but, of course, these would risk opening another field of controversy: the reluctance of many consumers to accept transgenic plants as viable alternatives to those developed through conventional breeding programs [38,39].

If natural plant products are to have a future in veterinary or human medicine, they must be developed to a stage where consistency of their effects can be assured, and rigorous quality control methods can be implemented. For this, knowledge of the active principles is essential, and specific *in vitro*-based assays that reflect accurately their anti-worm potential need to be available. Minimal side effects on either health or production traits are obvious requirements of any such products if they are to compete effectively with available drugs on the market.

## Medicinal fruits containing cysteine proteinases

Some of the earliest known medicinal anthelmintic plants include papaya (*Carica papaya*), figs (*Ficus *spp.) and pineapple (*Ananas comosus*). Anecdotal reports of their usage for the treatment of worm infections by the native inhabitants of Panama and South America stretch back to over a century ago [40]. Their extracts were shown to be highly effective in clearing the most obstinate of human intestinal worms, *Trichuris trichiura*, in the 1920s [41] and more effectively than any of the current synthetic drugs [42]. Indeed, European doctors used papain and papaya latex for the treatment of worms in the 19th century [[43,44] and see later in this review] but, it was not until the 1930s that they were shown to be actually capable of digesting nematodes [40] and their enzymic basis was discovered [45]. The active principles are now known to be cysteine proteinases (CP) that occur naturally in various parts of the plant, and Table 1 summarises some of those known to be contained within plants. For example, in pineapples, different combinations of enzymes occur in the stem, and in the fruit. The latex of both papaya and figs contains CPs.

**Table 1:** Plants which are known to contain cysteine proteinases with potential anthelmintic activity

Species	Enzymes known to be contained
Papaya	papain, chymopapain, caricain, glycyl endopeptidase
Fig	ficin, ficain
Pineapple	ananain, fruit bromelain, stem bromelain, comosain
Kiwi fruit	Actinidain
Egyptian milkweed	Asclepain
Cowhage	mucunain [108]

These plant-derived CPs probably evolved primarily to defend plants against insect pests [46] but possibly also against plant parasitic nematodes, against which they are likewise highly effective [47,48]. Among the other important roles of CPs are facilitating the coagulation of latex during the sealing of wounds on plants, leaf senescence and possibly also in the ripening process [49-51].

## Molecular characteristics of cysteine proteinases and their target proteins

The CPs are phylogenetically classified into 9 clans which show no evolutionary relationship between them, suggesting that the ability to utilise the thiolate anion of a cysteine residue as the nucleophile in peptide bond hydrolysis may have arisen independently on nine occasions. Fifty eight families of CPs are organised within these clans, and the CPs that have been shown to be anthelmintic are in Family C1, the papain family, in Clan CA [52,53]. Much is known about how these enzymes exert their activity. They have an active site groove into which the target polypetides bind. Lying across the groove are the active site cysteine and histidine residues, both crucial to the process of hydrolysis of the target polypeptide. The amino acid residues lining the groove determine which peptide bonds in a protein are susceptible to hydrolysis, by complementary binding with the substrate amino acid side-chains.

One important property of CPs is that their activity can be easily quantified. As was emphasized earlier, a useful property of any naturally occurring anthelmintic is to have an assay of its activity that is independent of the *in vivo *efficacy but correlates well with it. In the case of CPs, the specific inhibitor E-64 can be used to measure the operational molar concentration of enzyme activity in any preparation. For this assay, convenient substrates are also required and, for many of the enzymes, amide substrates with coloured or fluorimetric leaving groups can be used, such as, for papain, chymopapain and crude papaya latex, benzoyl-(D, L)-arginine-*p*-nitroanilide (Bz-Arg-pNA). On digestion, the nitroaniline group is released, which can be accurately quantified with the use of a spectrophotometer. For *F. carica *&* F. benjamina *latex, ficin, pineapple juice, kiwi fruit extract and *Asclepias sinaica *latex, a useful substrate is benzyloxycarbonyl-phenylalanyl-arginyl-*p*-nitroanilide (Z-Phe-Arg-pNA), and for stem bromelain, it is benzyloxycarbonyl-arginyl-arginyl-*p*-nitroanilide (Z-Arg-Arg-pNA). All these substrates are commercially available.

## Useful nematode screens for activity

Parasitic helminths have evolved to occupy virtually every conceivable niche in the mammalian host. Each species of intestinal nematode is a specialist to some degree and parasitizes a restricted region of the intestine. Table 2 is not an exhaustive list, but gives some examples of important nematode species affecting sheep, cattle, pigs, humans and rodents. Anthelmintic drugs are clearly likely to have varying efficacies in different regions of the gut because of the differences in physiology, including pH, enzyme content, fluid and solid content, etc., and therefore, in evaluating candidate drugs, it is important to assess their efficacies in laboratory systems with a spectrum of species that occupy different niches along the entire length of the intestine. The best suited systems for this approach are laboratory rodents, and, fortuitously, laboratory rodents are hosts to a range of relevant parasites, maintained in different laboratories throughout the world.

**Table 2:** Examples of nematode parasites that parasitize different regions of the intestinal tract in domestic animals, humans and rodents

Host		Species	
	Stomach	Small intestine	Large intestine

Sheep	Teladorsagia circumcincta Haemonchus contortus Trichostrongylus axei	Trichostrongylus colubriformis Nematodirus battus	Trichuris ovis
Cattle	Ostertagia ostertagi Trichostrongylus axei	Cooperia spp. Nematodirus spathiger	Oesophagostomum radiatum Trichuris globulosa
Pigs	Hyostrongylus rubidus	Ascaris suum	Oesophagostomum spp.
Humans		Ancylostoma duodenale Necator americanus Ascaris lumbricoides	Trichuris trichiura Enterobius vermicularis
Rodents	Protospirura muricola	Heligmosomoides bakeri Nippostrongylus brasiliensis Trichinella spiralis	Trichuris muris

Of the rodent species listed in Table 2, *Heligmosomoides bakeri *is perhaps the easiest to maintain and to work with. *H. bakeri *has changed its name twice in recent times, being formally known as *Nematospiroides dubius*, and then *H. polygyrus*, but now correctly as *H. bakeri *[54]. This species causes chronic infections in the anterior of the small intestine of mice, so there is little concern about loss of parasites through host immunity in the immediate weeks following a single-pulse infection [55]. With species such as *Trichinella spiralis *and *Nippostrongylus brasiliensis*, which are expelled within 3 weeks of infection by rats, experimental trials have to be carefully timed to complete observations before worm expulsion through immunity, otherwise it is not possible to distinguish between worm loss arising through treatment with a candidate drug and worm loss through the development of the host-protective immune response.

Many of the most devastating problems in ruminants are caused by nematodes that live in the abomasum, the fourth stomach of ruminants, but there are no ideal models of these species in rodents. Recently, a spirurid worm of wild spiny mice [56] has been isolated and adapted for passage through laboratory mice [57], and we have used this species successfully to assess the effects of our candidate anthelmintics in the rodent stomach [58]. Although it is a member of a phylogenetic group that is not closely related to the Trichostrongyloidae, it nevertheless lives in the acid environment of the stomach and presents candidate drugs with similar problems to those encountered in the abomasum of ruminants.

Many different species of nematodes exploit the large intestines of mammals and, in some hosts, such as horses, the list is very long indeed, notably the cyathostomins. In mice, *Trichuris muris*, which is closely related to the *Trichuris *spp. that infect humans (*T. trichiura*), dogs (*T. vulpis*), pigs (*T. suis*) and ruminants (*T. ovis, T. globulosa*), is an obvious species to exploit in screening programs.

## Comparison of the efficacy of different extracts on parasite motility *in vitro*

Note all experiments carried out by the authors involving the use of laboratory animals were approved by the University of Nottingham ethics committee and were carried out under UK Home Office Licence (Project Licence number 40/2621).

Most species of nematodes can be kept alive and motile in simple saline media for short periods of time after removal from their hosts. As an initial screen, we developed a straightforward motility assay which was simple to implement and highly reproducible. Worms were placed in a saline solution kept at 37°C on a laboratory bench incubator, and their motility was scored on a scale of 0–5, where a score of 5 represents a highly active motile worm, 4 slightly less active, 3 less active still, 2 lethargic but capable of independent movement, 1 movement only apparent when prodded, and 0 no movement even when prodded. With *H. bakeri *and *T. muris *the motility of worms kept in control saline declined from the outset but slowly relative to that of worms exposed to effective anthelmintic agents. So with these species, assessment of efficacy was based on the expectation that motility would decline faster when the worms were exposed to an effective agent. *P. muricola*, however, is far more robust and capable of moving vigorously in media for well over 24 h.

**Figure 1:**
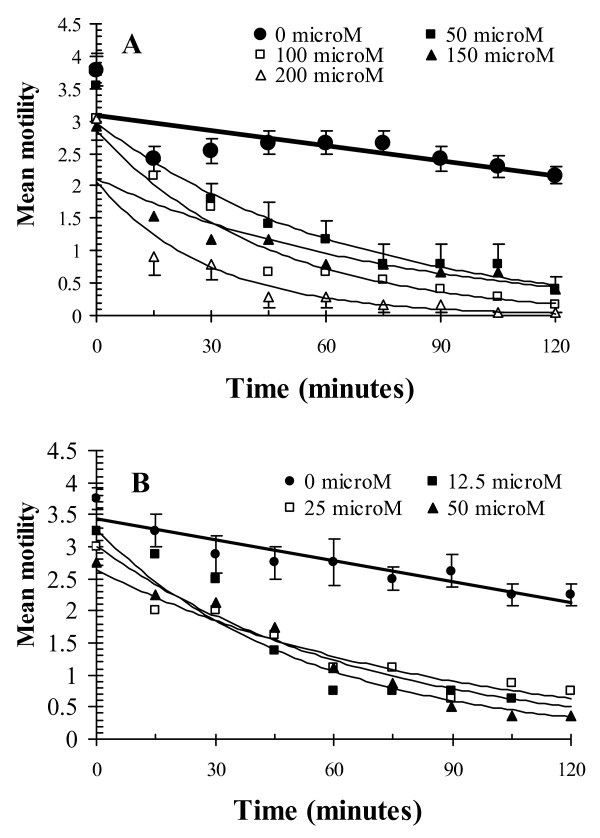
The effect of varying concentrations of enzyme activity in papaya latex (A) and in papain (B) on the motility of *Heligmosomoides bakeri in vitro*. Standard errors are given for the control (0 μM) groups and in selected treated groups only, to retain clarity. For the full statistical analysis of these and related data, see Stepek *et al*. [107]. Reprinted with permission.

Representative results are shown in Fig. 1, where the more rapid loss of motility of *H. bakeri *is clearly apparent when worms were exposed to increasing operational molar concentrations of CP activity. The dataset in Fig. 1B shows that all three concentrations of papain were highly effective in this case. Experiments such as these allow enzyme activity from different sources to be compared. Table 3 shows EC_50 _values extrapolated from these *in vitro *assays for plant extracts, and some purified enzymes as well. Many were extremely effective at micromolar concentrations, but interestingly kiwi fruit extract, containing actinidain, had no effect at all.

**Table 3:** Comparison of EC_50_s of a range of plant extracts known to contain cysteine proteinase activity

Enzyme	EC_50_(μM)
Milkweed latex extract	3
Ficin	5
Pineapple fruit extract	5
Chymopapain	7.5
Papain	7.5
Crude papaya latex	12.5
Stem bromelain	75
*Ficus benjamina* latex	140
*Ficus carica* latex	150
Kiwi fruit extract	None

All values are in micromolar concentrations of enzyme activity, as determined by active site titration, that caused 50% reduction in motility after 90 minutes of incubation with *H. bakeri*

**Figure 2:**
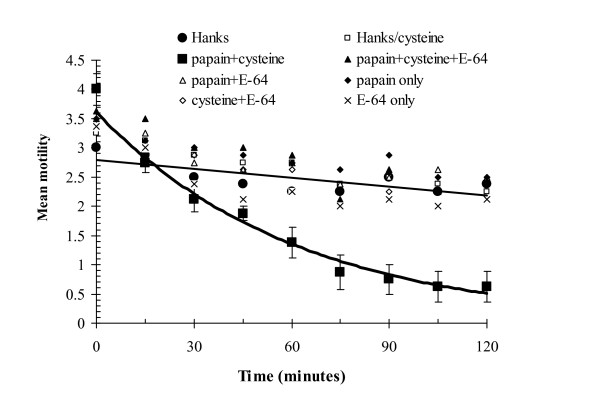
A factorial experiment to confirm that the effect on *H. bakeri *is mediated by cysteine proteinase activity. The worms were incubated in the presence/absence of papaya latex, presence/absence of cysteine, and presence/absence of the specific CP inhibitor E-64. The only treatment to show accelerated loss of motility was when worms were incubated with papaya latex, in the presence of cysteine but absence of E-64. This treatment is highlighted in bold. For the full statistical analysis of these and related data, see Stepek *et al*. [75]. Reprinted with permission.

An experiment following a factorial design, in which worms were incubated in the presence or absence of cysteine, presence or absence of papain, presence or absence of the specific CP inhibitor E-64, established firmly that the active ingredients were CPs. The only treatment in which worms showed accelerated loss of motility was incubation in the presence of exogenous cysteine and papain, but in the absence of E-64 (Fig. 2).

## Effects on the surface of the worms *in vitro*

**Figure 3:**
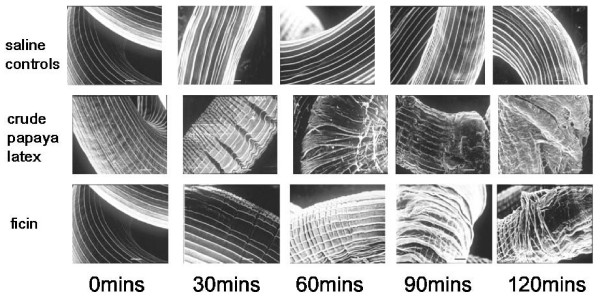
**Scanning electron micrographs of *Heligmosomoides bakeri *adult worms exposed to papaya latex and ficin *in vitro***. Clear evidence of damage to the cuticle can be seen from 30 min in 200 μM crude papaya latex and ficin, 30 μM. Note the transverse wrinkling leading to signs of shedding of the cuticle after 90–120 min. In contrast, worms incubated in Hanks's saline showed no sign of cuticular damage, even after 120 min incubation. Scale bar = 10 μm. For SEM preparation adult specimens of *Heligmosomoides bakeri *were fixed in glutaraldehyde, postfixed in osmium tetroxide, critical point dried and gold coated before examining in a Jeol JSM 840 scanning electron microscope. For further details see Stepek *et al *[107]. Reprinted with permission.

When exposed to CPs *in vitro*, all three species of nematodes (*H. bakeri*, *P. muricola *and *T. muris*) lost motility and, moreover, their surface began to show clearly apparent damage. Fig. 3 shows scanning electron micrographs of changes in *H. bakeri*, but similar changes were also seen in the other species. Most surprisingly, even the tough, resilient *P. muricola *could not hold out against the attack by CPs *in vitro *[58].

**Figure 4:**
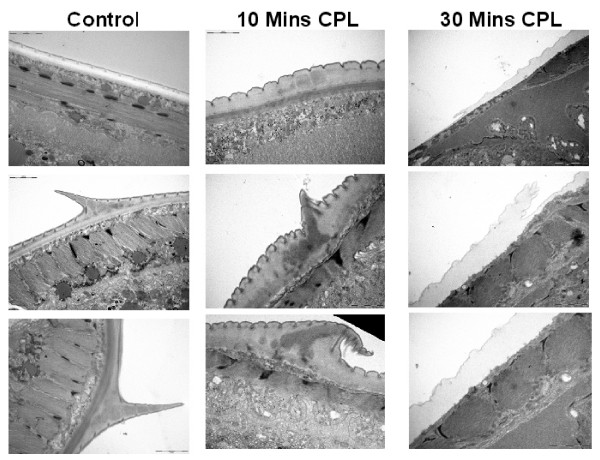
Transmission electron micrographs of the cuticle of adult *Heligmosomoides bakeri *exposed to crude papaya latex (CPL) (200 μM). After 10 minutes exposure the cuticle starts to exhibit swelling and wrinkling of the surface with the appearance of electron dense material and collapse of the cretes. Following 30 minutes of exposure to papaya latex much of the structure of the cuticle has been lost and detachment of the cuticle from the underlying hypodermis is apparent in places. For TEM preparation adult specimens of *Heligmosomoides bakeri *were fixed in glutaraladehyde, postfixed in osmium tetroxide, dehydrated in ethanol embedded in epoxy resin and sectioned. The sections were stained in lead citrate and uranyl acetate and examined in a Jeol 1010 transmission electron microscope.

Examination by transmission electron microscopy showed that soon after exposure, the outermost layer of the cuticle began to crinkle, and the ridges called crêtes (longitudinal cuticular ridges without an internal cuticularized supporting element [59]), lost their rigidity, gained electron dense interiors, and began to shrink (Fig. 4). Eventually, the whole of the architecture normally seen on the outside of the parasite, constituting its synlophe (a technical term for the whole complex of crêtes that together comprise the equipment through which the nematode attaches to host villi [59]), disappeared revealing underlying collagen fibres, and then, at the weakest point, the internal high hydrostatic pressure within the pseudocoelomic cavity caused the worms to burst open, often carrying externally sections of the intestine and gonads (See Additional file 1). Further incubation invariably resulted in the almost complete digestion of the cuticle of the worms. This sequence was evident on all three species examined by us, and also on the canine hookworm *Ancylostoma ceylanicum *[60]. Although the detail of our observations, under both scanning and transmission electron microscopes, on the changes taking place were original, the fact that parasitic worms dissolve in the presence of extracts from papaya, pineapple and figs is not itself new. It was first suggested in the late 1870s [61] and then shown convincingly in the last century, first with extracts of figs [40] and then pineapples [62] and papain on *Ascaris suum *[[63], cited as *A. lumbricoides *but obtained from pigs so more likely to be *A. suum*].

**Figure 5:**
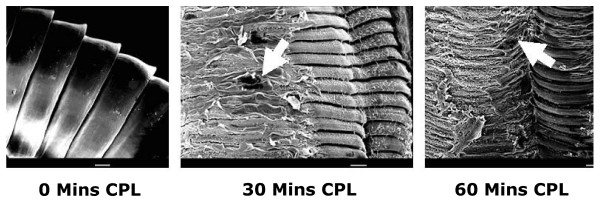
Scanning electron microscopy of the surface of *Rodentolepis microstoma *incubated with crude papaya latex (CPL) *in vitro*. The micrographs were taken at equivalent points along the worm surface, near the mid-point. The digestion of the tegumental surface was evident by 30 mins when the worms were incubated with 25 μM papaya latex. Lesions attributable to the activity of the CPs in papaya latex are highlighted by the arrows and the damage increased substantially between 30 and 60 mins after exposure. The tegument remained intact, with no visible damage, even after 2 hours, on incubation with Hanks' saline + 16 mM cysteine (not shown). Scale bar = 100 μm. (see reference [60]). Reprinted with permission.

Even more surprisingly, we found that the surface of the tapeworm *Rodentolepis microstoma *also showed major structural changes (Fig. 5), reflected in damage evident at the scanning microscopical level to the surface of the syncytial external layer of the tegument. These studies on the effects of CPs on cestodes are still at a very early stage, and, currently, we have little information on the details of the process involved. In fact, the anthelmintic properties of pineapple extract against helminths other than nematodes, was reported as early as 1939 by Berger and Asenjo [62] who found that the porcine acanthocephalan parasite *Macracanthorynchus hirundinaceus *was dissolved in the presence of fresh pineapple juice. Later, the same authors found that papain did not have the same effect against the acanthocephalan [63]. The observations that CPs actually damage cestodes, and acanthocepahalans, albeit selectively, raise the possibility that some CPs may have a wider spectrum of activity than purely against nematodes. Interestingly, de Amorin *et al*. [64], failed to detect an in *vivo *effect of *Ficus *latex on *Rodentolepis nana *in mice. Nevertheless, the idea that there may be common target sites in proteins in the nematode cuticle and the cestode and acanthocephalan teguments is intriguing.

## Effect of cysteine proteinases on worm burdens *in vivo*

The real test of whether CPs have potential for development as novel anthelmintics is the demonstration that they actually reduce worm burdens in infected animals.

### Effect on helminth infections in humans

Indications that papaya latex and papain are actually active in this respect go back to the early 1800s, when they were used by physicians in Europe for treatment of nematode infections [29] at a time when there were few alternatives available and the synthetic drugs had not yet been invented. Among other reports, Fernan-Nunez [65] reported a centuries-old custom of native people in Columbia who used the sap of the fig *F. laurifolia *(known locally as Leche de Higueron) as a treatment for worm infections, especially *Trichuris trichiura*. Two further reports from this period, that are not based on full publications but are cited by others, include the recovery of up to 500 expelled *T. trichiura *from subjects treated with the sap of *F. elastica *in Panama by Schapiro [66] and the effective use of "Leche de Higueron" by Hall [67] against trichiuriasis in Nicaragua. Caldwell & Caldwell [41] reported successful treatment of patients at the Scarcy Hospital for the insane at Mt. Vernon, Alabama where trichiuriasis was extensive, with Higuerolatex. This report documents faecal egg count reductions of up to 91%, and averaging 89.5%, exceeding quite markedly the average efficacy of modern anthelmintics [42] for *T. trichiura *infections. Higuerolatex was also found to be effective against *A. lumbricoides*, reducing faecal egg counts by 89.7%.

More recently, the commercially available formulation Vermizym, manufactured in Germany, (based on papain) was shown to be effective against human pinworm (*Enterobius vermicularis*) and round worm (*Ascaris lumbricoides*) infections in a trial involving 60 subjects, who were apparently completely cleared of worms [68]. Jonxis & Bekins [43], in Holland, used a preparation called Velardon, also based on papain, to treat children less than 8 years old for *Ascaris *infections. Stransky & Reyes [44], working in the Philippines, assessed Vermizyn, treating subjects with three doses at about one and a half hour intervals, each with a teaspoon of Vermizyn. They reported very high efficacy reflected in reduced faecal egg counts after treatment and expulsion of adult *Ascaris *from treated subjects. They also confirmed that *Trichuris *infections were markedly reduced but failed to detect any effect on hookworm infections. Another trial with the same product showed that it cured 70% of subjects with whipworm (*Trichuris trichiura*) infections [69].

A trial in Amazonia in the 1980s with the latex of *F. glabrata *(= *F. laurifloria*?, the ojé tree) indicated strong efficacy against *A. lumbricoides *and *Trichuris trichiura*, and even *Necator americanus *in humans [70], but the study did not meet contemporary standards for such trials and lacked the necessary statistical analysis.

### Effect on helminth infections in companion animals

There are few reports of the use of CPs to treat companion animals, but the successful use of the latex from papaya (at 1.3 ml/kg) to remove worms (most probably *Toxocara*, although reported as *Ascaris*) from dogs in Cairo was reported by Nagaty *et al.*, [71].

### Effect on helminth infections in livestock

Evidence that papaya latex was highly effective against *A. suum *in pigs was provided by Satrija *et al*. [72], but we are not aware of any other trials in monogastric livestock. To our knowledge, there is only one reference to the effect of CPs on parasites of ruminants, in which the animals in question were treated orally with papaya latex [73], but this publication is unavailable in the West.

### Effect on helminth infections in experimental rodent trials

Satrija *et al *[74] provided evidence that papaya latex was effective in removing *H. bakeri *from experimentally infected mice. The dose response reported in this paper indicated that a single treatment at 8 mg/kg body weight papaya latex caused an 84.5% reduction in worm burdens.

**Figure 6:**
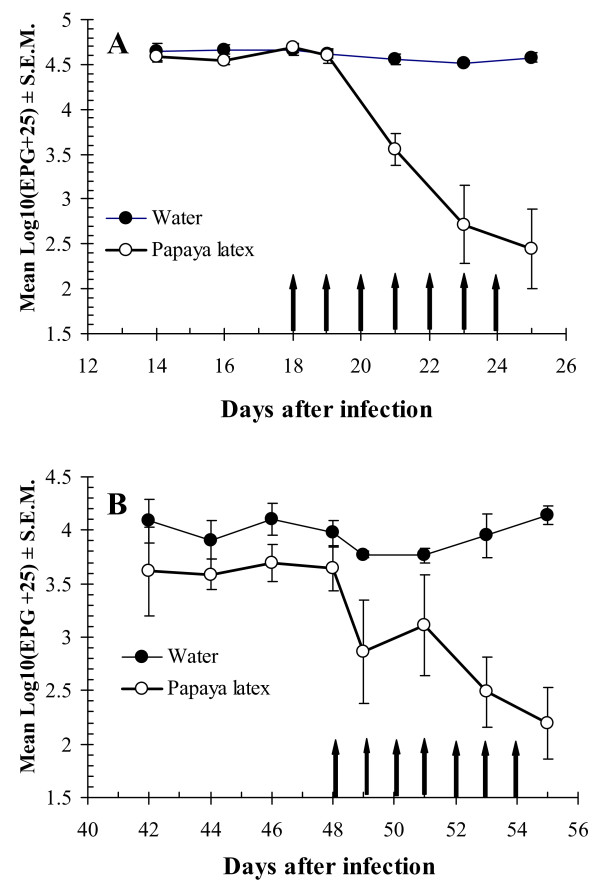
The effect of treatment with papaya latex on faecal egg counts in mice infected with 200 L3 of *H. bakeri *(A) or 100 eggs of *T. muris *(B). In some cases, the error bars do not show because they are very tight and obscured by the mean data point. In (A) treatment with papaya latex (133 nmol active cysteine proteinase/mouse/day) or water was initiated on day 18 and continued daily until day 24 [75]. In (B) the first dose of papaya latex (337 nmol active cysteine proteinase/mouse/day) or water was given on day 48 and continued daily until day 54 [76]. In both figures, the days of treatment are shown by vertical arrows on the abscissa. Reprinted with permission.

We gave mice infected with *H. bakeri *daily treatments of papaya latex for seven days, and assessed faecal egg counts (FEC) on three days before treatment and then on four days after treatment, including the final day when animals were culled for worm counts [75]. The data in Fig. 6a show that, soon after treatment with papaya latex commenced, faecal egg counts of *H. bakeri *fell, and, by day 25, FEC had fallen by 97% in the papaya latex-treated animals. When the animals were autopsied, the reduction in parasite burdens was 92%, although complete loss of worms was not achieved [75].

Fig. 6b shows that treatment of mice infected with *T. muris *also resulted in rapid reduction of FEC, mean values dropping by 98% by day 55 post infection [76]. Worm burdens fell by 93% relative to the control group treated only with water. These results demonstrate convincingly that at least some enzyme activity can survive passage through the upper intestine and that sufficient amounts are present in the large intestine to cause the suppression of egg counts and loss of *T. muris*. In this respect, it is pertinent that Hale [77] showed proteolytic activity throughout the alimentary tract and even in the faeces of mice that had been treated with bromelain, and these observations concur with our data showing that papaya latex CP activity is recoverable throughout the intestine of mice in the hours after treatment, but accumulates particularly in the large intestine [58]. These experiments provide independent confirmation of the outcomes of earlier trials in humans, with worms living in both the small and large intestines [68-70].

**Figure 7:**
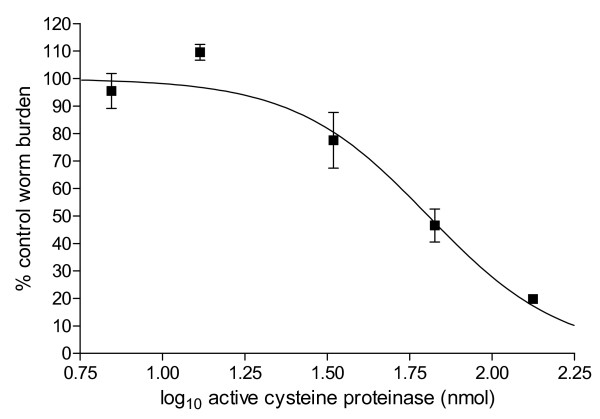
Dose response with papaya latex in mice infected with *H. bakeri*. Mice were infected with 200 *H. bakeri *and given varying doses of papaya latex corresponding to the nmol amounts of active enzyme illustrated in the figure. Worms were counted 25 days post infection. The log_10 _ED_50 _was calculated as 1.8 (95% CL = 1.72–1.89), corresponding to 67nmoles of active cysteine proteinase. The log_10 _ED_95 _was 2.4 (95% CL = 2.14–2.67), corresponding to 133nmoles of active enzyme. For full details and comprehensive analysis see, Stepek *et al. *[75]. Reprinted with permission.

**Figure 8:**
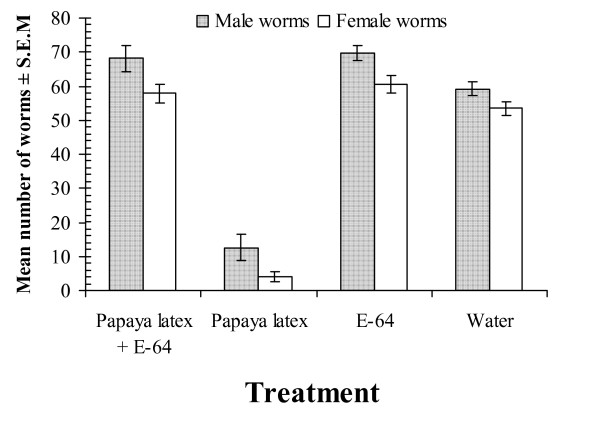
Evidence that the *in vivo *effect of papaya latex on *H. bakeri *is mediated through cysteine proteinases. Groups of mice infected with 200 L3 of *H. bakeri *were treated with water, E-64 alone, papaya latex (135 nmoles active enzyme) or papaya latex pre-incubated with E-64 (0.64 nM) for 15 minutes prior to oral delivery to mice. Only untreated papaya latex caused a significant reduction in worm burdens. For full analysis and further details, see Stepek *et al *[75]. Reprinted with permission.

Dose response experiments confirmed that the efficacy of papaya latex was based on low amounts of CP, with ED_50_s in the nanomolar range (Fig. 7). Such low amounts with the high anti-parasite activity that we observed indicate that much of the papaya latex is in fact non-active, and raises the possibility that refinement and concentration of the active principles will enable a reduction of the volumes and weights of papaya latex that have to be currently given to animals to cause loss of worms. Satrija *et al*. [74] administered milligram quantities to detect an effect on *H. bakeri *in mice and 4–8 g/kg body weight (total doses exceeding 150 g/animal) to cause a reduction in *Ascaris suum *in pigs, and our experience with *H. bakeri *concur with these values. However, our demonstration that the active principles are actually present in much smaller concentrations provides the clue that concentration of the active principles may be a worthwhile avenue for refining the use of these agents. Our unpublished data indicate that the active principles are soluble and found in the supernatant fraction of papaya latex after centrifugation, encouraging us to believe that further refinement is a feasible option. As with *in vitro *experiments, we were able to demonstrate that *in vivo *activity also depended on the presence of active CPs, since papaya latex that had been treated with E-64, had no effect on worm burdens and E-64 by itself was also without any effect on worms (Fig. 8), [75]).

A relevant question to resolve was whether the mode of action of CPs in papaya latex *in vivo *was the same as that evident *in vitro*. One complication of addressing this question adequately is that as soon as worms are damaged they are likely to begin moving down the intestine, since they would become incapable of holding their position against the flow of ingesta as it is pushed down the intestine by peristalsis. Thus damaged worms are likely to be detected outside their normal site in the gut and it could be argued that the damage was not so much a reflection of the activity of the CPs but rather the consequence of moving into an abnormal site in the gut. However, our experiments contained natural controls that helped to dispel this concern. Papaya latex does not clear all the worms out at each treatment, so some worms remain in their normal anterior site. Indeed we found that 1 hour after treatment, worms could be detected in the lower intestine, showing damaged cuticle and signs of erosion of their surface much like those observed *in vitro*. Two hours after treatment some worms were in the colon with even more substantial damage to their surface, showing complete erosion of the surface layers of the cuticle and exposure of the underlying collagen fibers. However, even at 3 and 4 hours after treatment other worms were seen in the upper small intestine, in their normal site, showing a normal appearance of the synlophe with an undamaged cuticle. That, together with our observation that after treatment with conventional anthelmintics (such as pyrantel, which paralyses worms), no changes were seen in the cuticle as the worms were retrieved from the lower intestine, indicate that it is the CP activity, and not translocation into abnormal sectors of the gut, that is responsible for the damage to worms *in vivo *[75].

## The special case of worms that parasitize the stomach

The CPs found in the latices and extracts of fruits all have a neutral pH optimum of around 7, with a range, for example in the case of ficin, in which some activity is retained from pH 4 to 8.5 [40]. Fruit derived, secreted CPs cannot work effectively at lower or higher pH (although some intracellular CPs have lower optimal pHs), so we were not expecting to see an immediate effect on our model stomach worm *P. muricola*. It has even been suggested that the effect of acidity on CPs is irreversible [78], but quite clearly that cannot be the whole story because in our experiments and the earlier trials with human parasitic worms, the anthelmintic activity was convincingly demonstrated against worms in the small intestine and in the colon [75,76]. As expected, we initially observed no effects against *P. muricola *when the papaya latex was given orally. However, when acid secretion was temporarily blocked by giving the antacid cimetidine 30 minutes before the papaya latex, we observed loss of most female worms, although surprisingly no effect on males [58]. This curious sex bias may just be a question of the available cuticular surface, since female worms are substantially larger than males [79], although the surface area/volume ratio is larger in smaller organisms. It may also reflect molecular differences in the structural composition of the cuticles of the two sexes of this species. The observation that CPs in combination with cimetidine can be made to express anthelmintic activity in a site where the pH would normally be too acidic for them to be able to work effectively, opens the door to other refinements and combination treatments, although, as yet, this is an unexplored topic.

## Limitations of plant derived CPs

In exploring the range of anthelmintic properties of CPs, we also encountered some limitations to their usefulness. For example, we found that they were without any significant effects either in terms of motility or actual evident cuticular damage to *Caenorhabditis elegans *(Stepek *et al*, unpublished observation). In some respects, this was a disappointing finding, since we had hoped that a rapid through-put assay could be developed to screen as yet untested plant sources for useful CP based anthelmintic properties. We even examined some surface cuticular mutant strains, but all to no avail (Stepek, Buttle, Lowe, Duce and Behnke, unpublished observations).

Papaya latex was also without any effect on the parasitic stages of nematodes that live in the mucosa as distinct from the lumen of the intestine. The early developmental stages of *H. bakeri *are located in the submucosa, often lying just underneath the serosa, and when treatment was given to coincide with this phase of infection, the worms were unaffected, maturing normally and emerging subsequently to live in the gut lumen [75]. Similarly, we found no effect on the early stages of *T. muris*, which burrow deep into the crypts in the large intestine [76]. In some respect, this may actually be a useful property of these agents since there is considerable concern about the development of resistance to the available synthetic anthelmintics, and the argument has been made that worms in refugia may help to offset the appearance of resistance and subsequent loss of activity [80,81]. Treatment with CPs would be selective in the sense that it would be stage-specific, therefore, damaging only those worms that actually expose parts of their surface in the gut lumen. Any developmental stage buried in the gut mucosa would escape treatment and help to repopulate the gut subsequently. This in turn would mean that treatment would need to be repeated frequently where the exposure to infective stages is high, such as among ruminants grazing contaminated pasture. However, the treatment would be directed at the adult stages that are seeding the pasture with eggs, and would therefore have an impact on transmission. On an even more positive note, it may be possible to modify feeding regimes to include CPs in the supplementary feed that is often given to grazing livestock to ensure that they have a balanced diet and do not lack essential ions and vitamins, particularly during pregnancy and lactation. An alternative strategy may be to provide fodder containing parts of pineapples, figs and papaya that are rich in naturally-occurring CPs. Such approaches are already being evaluated among small holder farmers keeping pigs, goats and cattle in Australasia [82,83].

We were surprised to find that the plant-derived CPs did not affect the motility or cuticle of free-living larvae (L1, L2 and L3) of *H. bakeri*, nor the development of larvae within eggs (Stepek *et al*., unpublished data). Given that *C. elegans *was not affected, this may indicate that free-living and soil-dwelling stages of parasitic nematodes, as well as totally free-living species, have a different composition to their outermost cuticular layers. CPs are not only found in plants; they are also secreted by some soil-dwelling bacteria and may be present in the soil [84] and hence, soil-dwelling nematodes may have evolved defences against CPs. On the other hand, the mammalian intestine does not secrete CPs, the key intestinal enzymes being either serine or aspartic proteinases, against which intestinal nematodes have defences [85,86]. In the absence of host secreted intestinal CPs, it is not unreasonable for parasitic stages to cease expression of defences against CPs since cystatins (inhibitors of CPs) would be largely superfluous but nevertheless metabolically demanding to manufacture. In a similar vane, the third stage larvae of *P. muricola*, which encyst in the haemocoele of insects, were also found not to be susceptible to CPs, and it is pertinent that the *Drosophila melanogaster *genome encodes 13 Family C1 proteases [87] perhaps suggesting that insect-parasitic larvae of nematodes may have evolved effective countermeasures as a consequence. Interestingly, some insects are susceptible to CPs from fruits, and indeed defence against insect pests is considered to be one of their essential functions [46,88]. This role is supported by the disappearance of the protective latex from fruits such as papaya once they have ripened, although pineapples are an obvious exception [89].

Although Satrija *et al *[73,90] failed to find a significant effect on the ruminant abomasal nematode *H. contortus *by oral delivery, we believe that alternative methods of administration, such as slow release from boluses, and combination treatments to temporarily suppress abomasal acidity, may succeed, but they have still to be evaluated. If useful efficacy against the intestinal parasites of ruminants were to be demonstrated convincingly, the industry may show some interest, since it is in their role in livestock agriculture that anthelmintics succeed or fail.

## Competition with synthetic anthelmintics and the scope for improving formulation and delivery

There can be little doubt that in an environment where available anthelmintic drugs are rapidly losing efficacy, alternatives have to be sought before the current drugs cease having any useful effect. There are already signs that in some parts of the world livestock farming has become almost impossible and, for example, farmers have abandoned sheep husbandry because anthelmintics could no longer be used to control worm burdens [91-93]. The published information on naturally occurring CPs raises optimism that they may be useful additions to the available armoury of weapons against GI helminths. Where synthetic anthelmintics are still effective, CPs would stand little chance in direct competition (more complex manufacture and preparation, and expense), but they may be a useful weapon where resistance has already tightened its grip. They may also provide a useful control method where CP-producing plants are indigenous in tropical and sub-tropical countries where poor pastoral communities are the norm and nematode infections are endemic. In such areas, farmers could conceivably grow their own anthelmintic crops. However, for commercial production to become a reality, there is still a long way to go in terms of provision of adequate supplies and improvement of formulation and delivery. The pharmaceutical laboratories around the world have invented many sophisticated and elaborate delivery systems for drugs, and we believe that it is not beyond the scope of possibilities for contemporary methods of formulation and delivery to be adapted to improve the access of CPs to the sites within hosts where their target helminths live.

From an economic standpoint, ruminants must be the prime target hosts, since this is where anthelmintics succeed or fail in terms of their market success and profitability. Formulations that will enable the CPs to pass through the rumen, show efficacy in the abomasum and then in the small and large intestines are not beyond the scope of achievable objectives, given current advances in pharmacy.

As explained earlier there is already strong evidence that CPs show efficacy in monogastric hosts such as rodents, pigs, humans and even poultry. To our knowledge, they have not been tested in horses, but resistance among intestinal nematodes of horses is a big problem in the racing and leisure industries, and there are concerns in both that resistance is becoming more prevalent [6,94].

Satrija *et al *[72] showed that papaya latex effectively eliminated *A. suum *from pigs, but were concerned about some of the side effects in animals given the higher doses which were more effective at clearing worms (see below for more on pathology). Although pigs have their own spectrum of helminths, including several species of nematodes (Table 2), there is little indication that these cause any significant economic losses on farms where the animals are raised intensively indoors [95]. However, there are reports that in developing countries pigs do harbour quite intense infections with nematodes and these may be responsible for losses in production efficiency [96,97]. There are also some indications of resistance [9], although this is not yet a serious problem in the pig industry worldwide. However, in developed countries there is now a strong trend for a return to outdoor, free-range pig husbandry, and free-range farming is undoubtedly going to lead to higher worm burdens since fields cannot be cleaned as easily or as frequently as indoor concrete pens [98]. Controlling worms through regular dosing of outdoor pigs is likely to see an exacerbation of resistance in the future.

As stated earlier, CPs, have been used as anthelmintics for humans in the past, even in Europe in the eighteenth century, but these and related products have never prospered commercially because GI nematode infections are not a real issue in Europe, and because they were overshadowed by the synthetic drugs [34]. However, there are indications that some species of human GI nematodes, notably hookworms, are beginning to show signs of resistance [12,15], and it may be that we will have to return to these older recipes in the future. Undoubtedly, the formulation and delivery of CPs for human use can be improved, and this may help to make them acceptable in modern medicine.

## Other uses

The molecular basis of the effect on nematodes is a fertile field for future exploration. Nothing is known about the target sites of CPs on the nematode cuticle. These must be proteins, but what precise role do they play in the cuticle and where exactly are they situated in relation to other cuticular molecules such that their digestion has immediate dramatic consequences? And what of tapeworms and acanthocephalans? Do they share the same surface proteins? This is unlikely given the fundamental differences in structure between the nematode cuticle, and the platyhelminth and acanthocephalan teguments, but even if the affected proteins are quite distinct with common target sites, that in itself will be of considerable interest. Can further research into these aspects help to improve our understanding of the composition of the nematode cuticle and the platyhelminth and acanthocephalan teguments?

## Side effects and pathology

The early reports of the use of papain for treatment of human infections generally found little or no evidence of side effects that could be ascribed as additional to the discomfort and pathology attributable to the worms. Jonxis & Bekius [43] treated children less than 8 years old and reported that none of the children showed any problems during and after treatment. Likewise, our experiments in mice [75], mostly with papaya latex, and those of Satrija *et al *[72,74], have failed to indicate any significant side effects, although the latter authors found some signs of constipation at the higher doses that they employed. However, the intestinal mucosa appeared not to have been affected and this concurs with our own data, where the treated mice showed a temporary period of weight loss during the treatment phase, but even this was not substantial. Others have reported more substantial side effects. Satrija *et al. *[72] were concerned about the effects on the gut of pigs treated at the higher dose regimens. De Amorin *et al *[64] observed hemorrhagic microfoci in the mucosa of mice treated with the latices of *F. insipida *and *F. carica*, and commented on lethargy in mice treated at the highest levels, but we have not observed anything resembling these symptoms in mice treated with papaya latex. If these side effects are confirmed in respect of particular preparations of CPs, it is our belief that appropriate formulations may be devised to reduce their effects on the host, whilst preserving their anthelmintic properties.

CPs are renowned for their allergenic properties [99]. For example, *Der p*I from the house dust mite, which is responsible for many cases of asthma and various types of allergies, is related to those from fruits such as papaya [100]. As far as we are aware, the immunogenic properties of orally administered fruit-derived CPs have only been examined by Hale [77], who detected relatively low levels of circulating bromelain specific IgG after 18 weeks of daily oral treatment with bromelain. Nevertheless, papain is known to be allergenic when inhaled, and precautions would need to be taken if individuals or animals were presensitized [29].

## Who will use and profit from naturally-occurring anthelmintics?

Whilst we believe that, with some investment, CPs could be developed into anthelmintics that would have a useful role in modern agriculture, in order to gain a licence for use in this capacity, they would have to be of medicinal quality. In other words, they would have to pass the standard randomised, double-blind, placebo-controlled trials, and this, we are confident, they can do in respect of humans and pigs.

However, there is another road. CPs are naturally-occurring proteins in food that is eaten by people and animals on a daily basis, and it may be that they will turn out to have a better future as cruder versions in food additives, perhaps in the niche market of organic farming. Whole fruits, stems, leaves and extracts may all find a role in this respect.

There is yet another group of potential users, among the small-holder farmers in developing countries, who are only too familiar with the losses caused by GI nematodes to their livestock [2]. Again, cruder versions of extracts from plants, or plants of local providence, may be the solution in these cases and, as pointed out earlier, such trials are already under way in Australasia.

For human use, the CPs would have to jump through the same legislative hoops as any other medicinal product for public use, and eventually they would need to be available in a tablet or syrup form, that is palatable. However, given the enormous, burgeoning market in the health industry, CPs may also find a place as herbal medicines on the shelves of health stores rather than on those of pharmacists, although there is much controversy and a fine line to tread with respect to what is medicine and what a health supplement. Acceptable and appropriate labelling of such products to ensure that they are not classified by health authorities as conventional medicines is all important.

The biggest challenge of all is to persuade the pharmaceutical industry to support the development of CPs as novel anthelmintics, but there are seemingly insurmountable obstacles. How can the problem of prior art be surmounted? There is already a lot of information in the public domain about the anthelmintic properties of CPs, as we have indicated in this review, and this presents an impediment to attempts to patent the agents, an essential step in protecting investments in development. There is no easy solution, but the fact that we have available natural products that are known to show strong anthelmintic properties in a world where parasitic worms continue to cause disease in human populations and losses to the livestock agriculture, and seemingly cannot find the support to develop them into effective medicines, speaks loudly about contemporary priorities.

Perhaps the most exciting aspect of the possibilities for CPs is the scope for developing new crops for farmers in the developing nations of this world [27]. Currently, only two species of fig latices have been examined, and yet the family of trees to which these belong is enormous. There are hundreds of species of figs that have yet to be tested, and many other plants also synthesize CPs [101-103]. Some are likely to harbour less, others more effective CPs. Recently, it has been discovered that highland papayas (*Vasconcellea *spp.), of which 21 species are known, contain considerably higher levels of CP activity, associated with more enzyme in the latex and have more constituent CPs than *C. papaya *[104]. Other known sources of CPs include the milkweed family (Asclepiadaceae), as for example *Calotropis procerca *[105,106] and *Asclepias sinaica *[107], although, unlike those of fruits, the latices of these plants also contain powerful toxins such as cardiac glycosides. Many more plant sources remain to be discovered, as shown by a recent survey of plants from Mali [103], which revealed 9 of ten plant species to express CP activity and particularly high levels in *Cissus qaudrangularis*, *Securidaca longepedunculus *and *Stylosanthes erecta*. Many of the source plants require a warm climate, so when suitable plant species are identified, and once the market for CPs grows, there will be opportunities for farmers to take on this new challenge, which in the end, may turn out to be more lucrative than current conventional crops.

Equally exciting is the possibility that through conventional breeding, scientists of the future may be able to develop new varieties of the existing species as well as of new species that have yet to be discovered, that will produce greater quantities of, or more effective, CPs. Where CPs are co-expressed with toxins, it may be possible through selective breeding to lose their toxicity without impairing the yield of CPs. Although controversial, the methodology of transgenesis is well established in this age, and offers a further approach to improving the outputs of CPs from plants [38].

Undoubtedly, there are serious obstacles ahead, and a long path to tread, but we believe that, in this current age, where despite all the attempts at control, parasitic nematodes are still widely distributed in human populations and livestock, we need to think ahead and prepare for the eventuality of all currently available synthetic anthelmintics failing in the near future. In this context, standby alternatives will have an important role to play, but more importantly we believe that, with investment in improving the delivery and formulations of available CPs, they can be developed into effective anthelmintics with roles to play both in the niche markets of organic farming, providing small holders throughout the developing world with easily accessible local remedies and perhaps eventually in modern intensive farming and medicine as well.

## Competing interests

The authors declare that they have no competing interests.

## Authors' contributions

JMB conceived the study, supervised the laboratory work and wrote the paper. DJB conceived the study, supervised the laboratory work and wrote the paper. GS carried out the laboratory work and wrote the paper. AL carried out the laboratory work and wrote the paper. IRD conceived the study, supervised the laboratory work and wrote the paper. All authors read and approved the final manuscript.

## Supplementary Material

Additional file 1 A single adult female living specimen of *Heligmosomoides bakeri *was mounted on a microscope slide in Hanks's saline and sandwiched beneath a glass coverslip supported on petroleum jelly. The worm was imaged using a Zeiss Axiovert 135TV inverted microscope and photographed using a Scion CFW 1310 M digital camera. A solution of 25 μM papain was introduced below the coverslip and images were captured on a PC using Streampix III time-lapse software at a frame rate of approximately 1 image every 3 seconds for 30 minutes. The video file was edited and exported as an mpeg running at 10 times the original speed. The file is titled "H. bakeri female papain.mpg" and initially shows the worm freely moving in the papain solution. After the animal forms a helical coil, a lesion appears on the left of the worm. This is followed by rupture of the worm and loss of the viscera through the rupture leading to the death of the parasite. Click here for file
